# Green Leaf Volatile-Burst in *Selaginella moellendorffii*

**DOI:** 10.3389/fpls.2021.731694

**Published:** 2021-10-27

**Authors:** Moena Tanaka, Takao Koeduka, Kenji Matsui

**Affiliations:** Graduate School of Sciences and Technology for Innovation, Yamaguchi University, Yamaguchi, Japan

**Keywords:** green leaf volatiles (GLV), hydroperoxide lyase, CYP74 enzymes, non-seed plants, *Selaginella moellendorffii* (Spikemoss)

## Abstract

Green leaf volatiles (GLVs) consist of six-carbon volatile aldehydes, alcohols, and their esters. They are formed from polyunsaturated fatty acids and are involved in the defense of plants against herbivores and pathogens. GLVs generally have low concentrations in intact healthy plant tissues, but the biosynthetic pathway to form GLVs is quickly activated by mechanical damage to tissues, an event called the GLV-burst. Most seed plants have the ability to implement GLV-burst; however, this potential in non-seed plants has not been extensively researched. In this study, we examined the GLV-burst capacity of monilophytes, lycophytes, and bryophytes, and confirmed that monilophytes and lycophytes showed substantial GLV-burst ability, while bryophytes did not, with a few exceptions. When the genome sequence of a model lycophyte, *Selaginella moellendorffii* was reviewed, 10 genes were found that showed high similarity with the non-canonical cytochrome P450 enzymes, CYP74s, specialized in oxylipin formation. Recombinant proteins expressed with *Escherichia coli* showed that one of them had the ability to encode allene oxide synthase, and another encoded hydroperoxide lyase (HPL), preferring linolenic acid 13-hydroperoxide, and it was inferred that this gene was responsible for GLV-burst in *S. moellendorffii*. Based on the phylogenetic tree constructed with CYP74s of non-seed and seed plants, we hypothesized that HPL was acquired independently in the lycophyte and seed plants through diversification of CYP74 genes.

## Introduction

Oxylipins are biological compounds formed from fatty acids and their esters through at least one step of dioxygenation reaction. They are synthesized *de novo* when cells are activated by mechanical trauma or by specific stresses but are generally not stored in healthy cells. Most oxylipins are signaling molecules that act as autocrine, paracrine, or even endocrine, and in certain cases of volatile oxylipins found in fungi and plants, they can mediate communication between organisms. The fatty acid substrate mainly used for oxylipin formation depends on the kingdom to which the organism belongs. Animals prefer arachidonic acid for the biosynthesis of prostaglandins and leukotrienes (Funk, 2001), whereas fungi normally use linoleic acid to form precocious sexual inducers and volatile eight-carbon alcohols (Brodhun and Feussner, 2011). The biosynthesis of jasmonates and green leaf volatiles (GLVs) in vascular plants is generally performed with the use of α-linolenic acid (Wasternack and Feussner, 2018), although arachidonic acid is more often used for oxylipin formation in bryophytes (Croisier et al., 2010).

**FIGURE 1:**
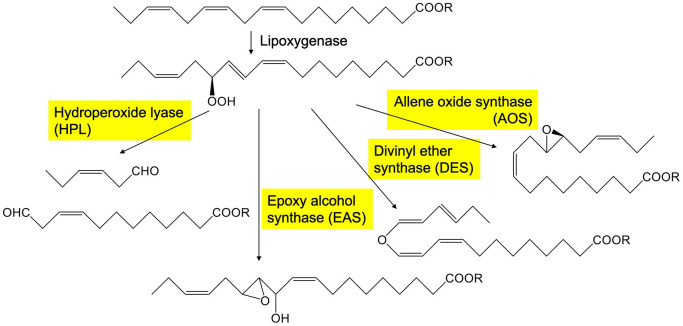
Representative pathway to form oxylipins from 13-hydroperoxide of linolenate catalyzed by CYP74s in plants. The enzymes with yellow background belong to CYP74 family.

Green leaf volatiles consist of six-carbon aldehydes, alcohols, and their esters (Matsui, 2006; Ameye et al., 2018). In intact and healthy, unstressed plant tissues, the GLV levels are generally low, but they are quickly formed when plant tissues are mechanically damaged (D’Auria et al., 2007; Mochizuki and Matsui, 2018). The free form of α-linolenic acid or the form esterified with glycerolipids is oxygenated by lipoxygenase to yield 13-hydroperoxides, which are further metabolized by the cytochrome P450 enzyme (CYP74B) fatty acid hydroperoxide lyase (HPL) (Figure 1). HPL forms (*Z*)-3-hexenal and 12-oxo-(*Z*)-9-dodecenoic acid from the 13-hydroperoxide of α-linolenic acid. The enzymatic conversion of α-linolenic acid (and its esters) quickly progresses when the cells are damaged by mechanical wounding from herbivores or pathogens. A portion of the (*Z*)-3-hexenal formed in the injured tissues diffuses out to the neighboring intact tissues, where it is efficiently reduced by cinnamaldehyde and hexenal reductase (CHR) to yield (*Z*)-3-hexen-1-ol (Tanaka et al., 2018), which is further converted into (*Z*)-3-hexen-1-yl acetate by acetyl CoA:(*Z*)-3-hexen-1-ol acetyltransferase (CHAT) (D’Auria et al., 2007).

This quick formation of GLVs, termed the GLV-burst in this study, is detectable within seconds after mechanical wounding of Arabidopsis leaves (D’Auria et al., 2007). GLVs are directly and indirectly involved in plant defense against herbivores and pathogens (Matsui, 2006; Ameye et al., 2018). Depletion of HPL in potato plants increases aphid performance (Vancanneyt et al., 2001). Overexpression of the HPL gene in *Arabidopsis* plants enhances defense against the necrotrophic fungal pathogen *Botrytis cinerea* (direct defense) and promotes attraction of parasitic wasps to herbivores feeding on the transgenic *Arabidopsis* leaves (indirect defense) (Shiojiri et al., 2006). To efficiently exert these defensive effects, rapid formation of GLVs is desirable.

The aim of this study was to determine when plants acquired GLV-burst ability. A comprehensive survey of 37 plant species, including bryophytes, monilophytes, and spermatophytes, showed that almost all vascular plants formed GLVs after disruption of the green tissues; however, one bryophyte, *Pogonatum inflexum*, revealed little GLV formation after damage (Hatanaka et al., 1978). The survey was furthered by examining 23 species of mosses collected in Switzerland and Germany (Croisier et al., 2010), most of which showed vigorous activity to form 1-octen-3-ol, but presented negligible GLV formation after freeze-thaw treatment, except for two species (*Neckera complanate* and *Dicranum scoparium*). HPL genes have been identified and studied in various seed plants (Matsui, 2006; Ameye et al., 2018), whereas there is only one report on the HPL gene in a non-seed plant, and that was from the moss *Physcomitrella patens* (Stumpe et al., 2006). This HPL (PpHPL) is largely involved in the formation of nine-carbon volatiles from linoleic acid 9-hydroperoxide and arachidonic acid 12-hydroperoxide (Stumpe et al., 2006); thus, its involvement in GLV-burst is implausible. Previously, we analyzed the genome sequences of *Marchantia polymorpha* and *Klebsormidium nitens* (formerly *K. flaccidum*), and revealed two and one CYP74 genes, respectively, all of which encoding allene oxide synthases (AOSs) but not HPL (Koeduka et al., 2015).

AOS is an enzyme that shares the substrate with HPL and converts linolenic acid 13-hydroperoxide into an unstable allene oxide (Figure 1), which when acted on by allene oxide cyclase is converted into 12-oxo-phytodienoic acid, which is further metabolized to yield jasmonic acid (Wasternack and Feussner, 2018). AOSs also belong to the CYP74 family and have high sequence similarity with HPLs. CYP74s are non-canonical cytochrome P450 enzymes that use hydroperoxides as opposed to molecular oxygen, which is characteristically used by canonical cytochrome P450 enzymes. CYP74s are almost exclusively found in plants (Brash, 2009). In addition to HPL and AOS, divinyl ether synthase (DES) and epoxyalcohol synthase (EAS) (Figure 1) belong to the CYP74 family with high sequence similarity. The enzymes grouped in the CYP74 family are quite similar to each other, and small amino acid exchange between them is often enough to interconvert their enzyme function (Lee et al., 2008; Toporkova et al., 2008, 2019; Scholz et al., 2012).

The ability of GLV-burst had likely been acquired between bryophytes and monilophytes, namely lycophytes, through innovation of the HPL that forms (*Z*)-3-hexenal as one of the products, by modifying the CYP74 genes available at that time. We collected several species of lycophytes, monilophytes, and bryophytes, and examined their GLV-burst ability. We also used the genome sequence of *Selaginella moellendorffii*, a lycophyte that has revealed a strong GLV-burst capacity. *S. moellendorffii* has 10 CYP74-like genes, six of which have been characterized as AOS, DES, or EAS (Gorina et al., 2016; Pratiwi et al., 2017; Toporkova et al., 2018). After examining the remaining four genes, we found that at least one of them encoded HPL and could be responsible for the GLV-burst. Based on the results shown in this study, the manner in which the plant lineage evolved the GLV-burst ability is discussed.

## Materials and Methods

### Plant Materials

*Selaginella moellendorffii* (provided by Dr. Xiaonan Xie, Utsunomiya University, Japan) was cultivated in a growth chamber at 22°C under 14 h of light/day (fluorescent lights at 62.5 μmol m^–2^ s^–1^) in normal potting soil mixed with Akadama and Hyuga soils (TACHIKAWA HEIWA NOUEN, Tochigi, Japan) in the ratio of 1:1:1. *Physcomitrella patens* (Gransden2004, provided by Prof. Mitsuyasu Hasebe, National Institute for Basic Biology, Japan) were grown in Jiffy pots (SAKATA SEED, Kanagawa, Japan) in a plant box (As One, Osaka, Japan). *Marchantia polymorpha* males (Takaragaike-1) were grown on half-strength Gamborg B5 medium (pH 5.5) with 1.0% agar at 22°C under continuous white light (fluorescent lights at 35 μmol m^–2^ s^–1^). *Sphagnum palustre* (purchased from a local market) was grown on peat moss (SAKATA SEED, Kanagawa, Japan) in the same growth chamber as *S. moellendorffii*. The list of plants used in this study is shown in Supplementary Table 1, and their phylogenetic relationship is illustrated in Supplementary Figure 1.

### Volatile Analysis

Plant leaves or thalli (100 mg fresh weight) were ground with 2 mL of 50 mM MES-KOH (pH 6.3) for 1 or 5 min with a mortar and pestle. The enzyme reactions were terminated by the addition of 2 mL of methyl *tert*-butyl ether containing 0.001% butylated hydroxytoluene and 5 nmol mL^–1^ tetralin (internal standard). After centrifugation, the resultant green supernatant was directly subjected to GC-MS analysis. To determine the amounts in intact tissues, the tissues were frozen in liquid nitrogen immediately after harvest and powdered with a Micro Smash MS-100R cell disruptor (TOMY, Tokyo, Japan) with stainless steel beads (1 mm). The volatiles in the frozen powder were immediately extracted with the solvent containing the internal standard. The volatiles were analyzed using GC-MS (QP-2010, Shimadzu, Kyoto, Japan) with a DB-WAX column (30 m length × 0.25 mm diameter × 0.25 μm film thickness, Agilent Technologies, Santa Clara, CA, United States). Injection was performed using a splitless mode with a sampling time of 1 min at 240°C. A column temperature of 40°C was held for 5 min and increased by 5.0°C min^–1^ to 200°C. The carrier gas (He) was delivered at 44.8 cm s^–1^. The MS was operated in electron ionization mode with an ionization energy of 70 eV, and the temperatures of the ion source and interface were 200 and 240°C, respectively, with a continuous scan from *m/z* 40–350. For quantification, calibration curves were constructed with (*Z*)-3-hexenal (provided by Zeon Co., Tokyo, Japan), (*E*)-2-hexenal, and *n*-hexanal (both from FUJIFILM Wako Pure Chemical Co., Tokyo, Japan).

In order to compare volatiles in intact and partially wounded tissues, an SPME (solid phase micro extraction) fiber (50/30-μm DVB/Carboxen/PDMS; Supelco, MilliporeSigma, Burlington, MA, United States) was used essentially as described previously (Matsui et al., 2012; Tanaka et al., 2018). In brief, 150–200 mg fresh weight of shoots and roots of *S. moellendorffii* were left intact or cut into pieces (1–2 mm wide) with scissors and immediately placed in a glass vial (22 mL, Perkin Elmer, Waltham, MA, United States). The vial was sealed tightly with a butyl stopper and a crimp-top seal. The SPME fiber was exposed to the headspace of the vial for 30 min at 25°C. Thereafter, the fiber was inserted into the insertion port of the GC-MS system shown above but with the SPME Sleeve (Supelco) for the glass insert. The sampling time was 1 min with the splitless injection mode. The fiber was held in the injection port for 10 min to fully remove compounds from the matrix. Chromatography was carried out as shown above.

### Expression of Recombinant Enzymes in *Escherichia coli*

Total genomic DNA was extracted from the aboveground parts of *S. moellendorffii* and used as the template for PCR with primers designed based on the genome sequence in Phytozome^1^. Primers used in this study are listed in Supplementary Table 2. The resultant product was cloned into the expression vector pET100/D-TOPO (Invitrogen, Waltham, MA, United States) using the TOPO cloning strategy. *Escherichia coli* BL21 Star (DE3) was used as the host for the expression of recombinant proteins. An overnight culture (500 μL) of the cells was added to 300 mL of fresh LB with ampicillin (100 μg mL^–1^) and incubated at 37°C to an OD600 of 0.6. Isopropyl β-D-1-thiogalactopyranoside and 5-aminolevulinic acid (Cosmo Bio, Tokyo, Japan) were added to the cultures at 1 and 20 mM, respectively. After additional culture for 24 h at 16°C, the cells were harvested by centrifugation and disrupted with a sonicator (ULTRASONIC DISRUPTOR, TOMY, Tokyo, Japan) in 50 mM Tris–Cl (pH 8.0) buffer containing 1 mM EDTA, 100 mM NaCl, 0.4 mM phenylmethylsulfonyl fluoride, and 0.27 mg mL^–1^ lysozyme (FUJIFILM Wako Pure Chemical Co.). The lysate was centrifuged at 5,000 rpm for 5 min, and the resultant supernatant was re-centrifuged at 50,000 rpm (CS100FX, Eppendorf Himac, Ibaraki, Japan) for 60 min to obtain the membrane fraction, which was then resuspended in 50 mM Tris-Cl (pH 8.0) buffer containing 1 mM EDTA and 100 mM NaCl. BL21 Star (DE3) harboring pET15b (Takara Bio, Shiga, Japan) was used as the empty vector control.

### Enzyme Assay and Product Analysis

Hydroperoxide lyase activity was measured by following the decrease in absorption at 234 nm. The *E. coli* membrane fraction was mixed separately with 30 μM each of (9*Z*,11*E*,15*Z*)-13-hydroperoxyoctadeca-9,11,15-trienoic acid (13HPOT), (10*E*,12*Z*,15*Z*)-9-hydroperoxyoctadeca-10,12,15-trienoic acid (9HPOT), (9*Z*,11*E*)-13-hydroperoxyoctadeca-9,11-dienoic acid (13HPOD), and (10*E*,12*Z*)-9-hydroperoxyoctadeca-10,12-dienoic acid (9HPOD) in 50 mM MES-KOH (pH 6.0) at 25°C. The HPO substrates were prepared from α-linolenic acid and linoleic acid (MilliporeSigma) with soybean lipoxygenase-1 partially purified from soybean seeds and *Magnaporthe salvinii* lipoxygenase provided by Novozyme Japan (Chiba, Japan) as described previously (Koeduka et al., 2015; Sugio et al., 2018). The compositions of HPOs were analyzed using the straight-phase HPLC system after reducing the hydroperoxide group to hydroxide with triphenylphosphine (Sugio et al., 2018). The contamination of the positional and geometrical isomers in each HPO preparation was mostly negligible except for 9HPOT, which contained 26.2% of 13HPOT (Supplementary Figure 2).

To determine HPL activity, the initial velocity of decrease in absorption at 234 nm was followed for 10 s using an absorption coefficient of 25 mM^–1^ cm^–1^. The volatile products were analyzed by GC-MS as described above after extraction with methyl *tert*-butyl ether. For analyses of non-volatile products with LC-MS/MS, the reaction was terminated by the addition of 1 mL of methanol. The mixture was centrifuged at 2,000 rpm for 10 min. The resultant supernatant was directly used for LC-MS/MS (3200 Q-TRAP LC-MS/MS System, AB Sciex, Framingham, MA, United States, equipped with a Prominence UFLC, Shimadzu, Kyoto, Japan). The products were separated on a Mightysil RP18 column (150 mm × 2 mm inner diameter) with a binary gradient consisting of water/formic acid (100:0.1, v/v, solvent A) and acetonitrile/formic acid (100:0.1, v/v, solvent B). The run consisted of a linear increase from 20% B to 95% B over 30 min at a flow rate of 0.2 mL min^–1^. Compounds were detected with a photodiode array detector (SPD-M20A, Shimadzu) and by MS/MS using the negative ion mode with ion spray voltage −4,500 V, nitrogen as both the curtain gas (set to 40 arbitrary units) and collision gas (set to “high”), collision energy −10 V, scan range *m/z* 100–500, scan speed 4,000 Da s^–1^, and declustering potential −30 V. Recombinant CaHPL was expressed with *E. coli* and purified as described previously (Matsui et al., 1996). The 12-Oxo-(*Z*)-9-dodecenoic acid and 12-oxo-phytodienoic acid were purchased from Larodan AB (Solna, Sweden).

### Quantitative Real-Time RT-PCR Analysis

For mechanical wounding the shoots and roots were cut into pieces (1–2 mm long) with scissors, and placed on a sheet of wet paper towel at 25°C. Total RNA was isolated using the Qiagen RNeasy Plant Mini Kit according to the manufacturer’s instructions. Total RNA (0.25 μg) was then reverse transcribed with 2.5 μM aliquots of oligo(dT)_15_ primer (Invitrogen) and ReverTra Ace (derived from Moloney murine leukemia virus reverse transcriptase; Toyobo, Osaka, Japan) according to the manufacturer’s instructions. Real-time quantitative PCR was performed with the StepOne System (Thermo Fisher Scientific, Waltham, MA, United States). Ct values for the genes of interest were normalized to means of the reference gene for the ubiquitin gene (*XP_002966475*). Expression levels were calculated as relative amounts by using relative standard curves. The lowest value in each experiment was set at 1. Primers for RT-PCR are shown in Supplementary Table 2. Under the current RT-PCR condition SmHPL1a and SmHPL1b were indistinguishable.

### Phylogenetic Tree

Phylogenetic analyses were carried out using the Maximum Likelihood method based on the LG model + G + I in MEGAX (Kumar et al., 2018). Amino acid sequences were aligned using MAFFT v7.475 and Gblocks Server v0.91b. Genbank accession numbers of each CYP74 protein used for phylogenetic analysis are listed in Supplementary Table 3.

### Statistics

All values are presented as the mean ± SEM in triplicate at minimum. Statistical analyses were performed using Excel Toukei in Microsoft Excel (Social Survey Research Information Co., Tokyo, Japan). The statistical methods used are described in the figure captions.

## Results

### Distribution of Green Leaf Volatile-Burst Ability Among Monilophytes, Lycophytes, and Bryophytes

**FIGURE 2:**
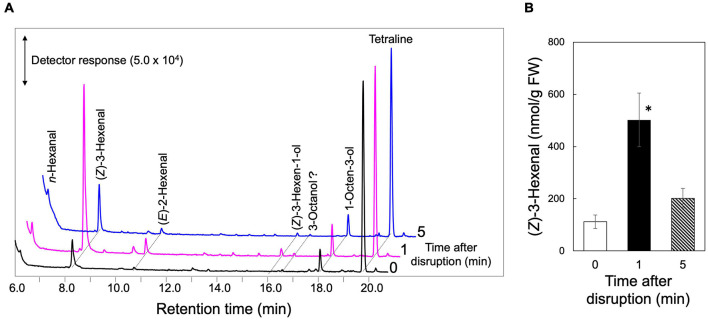
GLV-burst with *S. moellendorffii*. The upper ground parts of *S. moellendorffii* were ground in a mortar and pestle. After 1 and 5 min, the volatiles were extracted for GC-MS analyses. Intact leaves were also analyzed and shown at 0 min after disruption. **(A)** Representative chromatograms of volatiles found in the extracts. **(B)** The amounts of (*Z*)-3-hexenal found in the extracts. Mean values ± SE are shown (*n* = 3, biological replicates). Asterisk shows significant differences (one-way ANOVA, Tukey, *P* < 0.05).

As one of the representative plant species in the missing link between bryophytes and monilophytes, we focused on a lycophyte, *S. moellendorffii*, because of the availability of genome resources of this species (Banks et al., 2011; Ferrari et al., 2020). The leaves were completely ground in a mortar, and the volatile compounds formed in the homogenate were analyzed by GC-MS after 1 and 5 min of grinding (Figure 2). Small amounts of *n*-hexanal, (*Z*)-3-hexenal, (*E*)-2-hexenal, and 1-octen-3-ol were detected in the intact leaves as found with intact Arabidopsis leaves and *Nicotiana attenuata* leaves (Scala et al., 2013; Mochizuki et al., 2016; Joo et al., 2018), and, apart from 1-octen-3-ol, their levels increased substantially after 1 min of milling, which is a typical feature of the GLV-burst (D’Auria et al., 2007; Mochizuki and Matsui, 2018). Terpene compounds detected in stressed *S. moellendorffii* plants (Li et al., 2012) were not detected here. (*Z*)-3-Hexen-1-yl acetate, which is generally identified in partially wounded angiosperm leaves (Matsui et al., 2012) was not found in the completely disrupted *S. moellendorffii* leaves in our study. (Z)-3-Hexenyl acetate was not detected even with the partially damaged leaves (Supplementary Figure 3).

**FIGURE 3:**
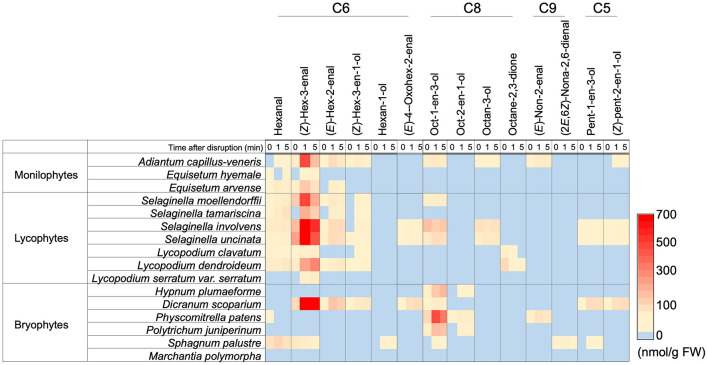
Distribution of the ability to form oxylipin volatiles among a subset of monilophyte, lycophyte, and bryophyte plant species. The amount of each compound is shown with color. The detailed values are shown in Supplementary Dataset 1.

The GLV-burst abilities were evaluated with the leaf or thalli of three species of monilophytes, seven species of lycophytes, and six species of bryophytes with GC-MS analyses after disruption of the organs, and the results are shown as a heat map (Figure 3, each result is summarized in Supplementary Dataset 1). As reported previously (Hatanaka et al., 1978), all plant species belonging to monilophytes showed substantial GLV-burst abilities, and lycophytes also showed capacity. In contrast, GLVs were not detected in four species of bryophytes, whereas *Dicranum scoparium* revealed a high propensity to form (*Z*)-3-hexenal, (*E*)-2-hexenal, (*Z*)-3-hexen-1-ol, and 4-oxo-(*E*)-2-hexenal. Similar observations have been reported for *D. scoparium* samples from Europe (Croisier et al., 2010). The intact thalli of *Sphagnum palustre* contained substantial amounts of *n*-hexanal and (*Z*)-3-hexenal, but their amounts scarcely increased after disruption of the thalli. The bryophytes, except for *Marchantia polymorpha*, formed a substantial amount of 1-octen-3-ol after grinding of their thalli, as reported (Croisier et al., 2010).

### Gene Mining

The results shown above suggest that GLV-burst ability was acquired when lycophytes diverged from the first vascular plants. The acquisition or development of gene(s) encoding HPL could account for the procurement of the ability to conduct the GLV-burst. We have previously determined that all the CYP74 genes found in the *M. polymorpha* and *Klebsormidium nitens* genomes encoded AOSs (Koeduka et al., 2015), and that no gene-encoding HPL has been identified in the liverwort and chalophyte to date. PpHPL found in *Physcomitrella patens* is specialized for the formation of nine-carbon volatiles such as (*E*)-2-nonenal; therefore, its involvement in the GLV-burst is excluded. As *S. moellendorffii* has been established as a model lycophyte (Weng and Noel, 2013) that has shown a substantial GLV-burst ability (Figure 2), we examined the genome sequence (Banks et al., 2011) and the expression atlas of *S. moellendorffii* (Ferrari et al., 2020) to mine CYP74 genes that could encode HPL.

Using BLASTP search on the *S. moellendorffii* v1.0 proteome database in Phytozome^2^ with AtHPL (At4g15440) as a query, we found more than 10 CYP74-like genes with E-values less than 2.4 × 10^–87^ (Supplementary Table 4) as reported previously (Koeduka et al., 2015). Among these, four genes have been confirmed as SmAOS2, SmDES1, SmDES2, and SmEAS1 through analysis of the catalytic activities of the corresponding recombinant proteins expressed in *E. coli* (Gorina et al., 2016; Pratiwi et al., 2017; Toporkova et al., 2018). The other two genes highly homologous to SmAOS2 were tentatively assigned as SmAOS1 and SmAOS3 (Pratiwi et al., 2017). The expression profile search in the CoNekT database^3^ constructed by a comprehensive gene expression analysis with *S. moellendorffii* (Ferrari et al., 2020) showed that, among the remaining four genes, Smo133317 (SmCYP74J1) and Smo92382 (SmCYP74L1) showed substantial expression in the aerial parts of *S. moellendorffii*, while the other two genes, Smo98717 (CYP74L2) and Smo413157 (CYP74L3), were expressed specifically in the roots (Supplementary Figure 4). The volatile analyses with intact and partially wounded roots and shoots indicated that the GLV-burst was predominantly detected in the green aerial parts (Supplementary Figure 3); therefore, we assumed that the genes expressed in the green part of the plant were involved in the GLV-burst. Accordingly, we focused on Smo133317 (SmCYP74J1) and Smo92382 (SmCYP74L1).

### Smo133317 (SmCYP74J1) Encodes 13AOS

**FIGURE 4:**
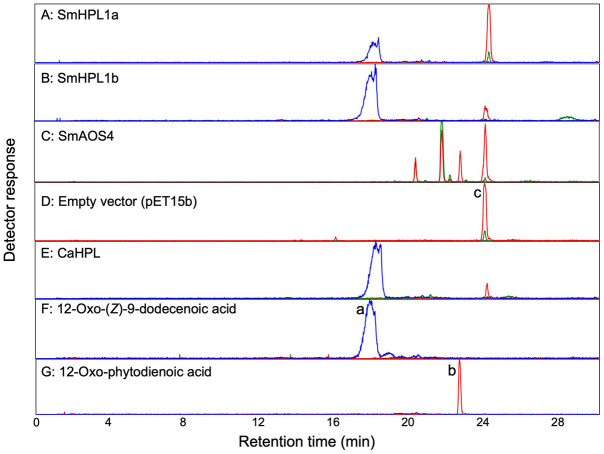
LC-MS/MS analyses of products formed by **(A)** recombinant SmHPL1a, **(B)** recombinant SmHPL1b, **(C)** recombinant SmAOS4, **(E)** recombinant bell pepper HPL (CaHPL) from 13HPOT. As the empty vector control, the membrane fraction of the *E. coli* cells harboring pET15b was reacted with 13HPOT **(D)**. Authentic standards of 12-oxo-(*Z*)-9-dodecenoic acid (peak a) **(F)** and 12-oxo-phytodienoic acid (OPDA) (peak b) **(G)** were also analyzed. The substrate, 13HPOT, is shown with peak c. The red trace shows extracted ion chromatograms with *m/z* of 291.2 ± 0.5 for hydroperoxides of linolenic acid [M-H_3_O^+^]^–^, 12-oxo-phytodienoic acid [M-H^+^], or colnelenic acid [M-H^+^]^–^. The blue trace is with *m/z* of 211.1 ± 0.5 for 12-oxo-(*Z*)-9-dodecenoic acid [M-H^+^]^–^. The green trace is with *m/z* of 309.2 ± 0.5 for hydroperoxides of linolenic acid [M-H^+^]^–^, α- and γ-ketols [M-H^+^]^–^, or epoxy alcohol [M-H^+^]^–^.

When Smo133317 (SmCYP74J1) was expressed in *E. coli* cells, the activity to degrade 13HPOT was detected in the membrane fraction prepared from the *E. coli* lysate through monitoring by following the decrease in absorption at 234 nm. GC-MS analysis of the products showed the formation of a trace amount of (*Z*)-3-hexenal that was equivalent to that of the reaction mixture with the heat-denatured membrane fraction. LC-MS/MS analysis indicated the formation of 12-oxo-phytodienoic acid (OPDA), 13-hydroxy-12-oxo-(9*Z*,15*Z*)-octadecadienoic acid (α-ketol), and 9-hydroxy-12-oxo-(10*E*,15*Z*)-octadecadienoic acid (γ-ketol) in the reaction of 13HPOT with the membrane fraction (Yanagi et al., 2011; Figure 4). No other peak corresponding to divinyl ether (*m/z* of 291.2) or epoxy alcohol (*m/z* of 309.2) that would be formed *via* DES or EAS activity on 13HPOT was detected. Accordingly, we named Smo133317 (SmCYP74J1) as SmAOS4. Recombinant SmAOS4 showed the highest activity at pH 5.5. Under representative reaction conditions with 40 μM of the substrate, the recombinant SmAOS4 showed the highest activity with 13HPOD, followed by 13HPOT (Table 1), whereas 9HPOD showed only 14% activity with 13HPOD.

**TABLE 1:** Substrate specificities of recombinant SmHPL1b and SmAOS4.

	SmHPL1b	SmAOS4
	
Substrate	Relative activity (%)	Relative activity (%)
13HPOT	100	52.6 ± 2.63
13HPOD	6.10 ± 1.22	100
9HPOT	2.18 ± 0.98	14.9 ± 0.88
9HPOT	2.32 ± 0.85	14.0 ± 0.98

### Smo92382 (SmCYP74L1) and Its Variant Encode 13HPL

**FIGURE 5:**
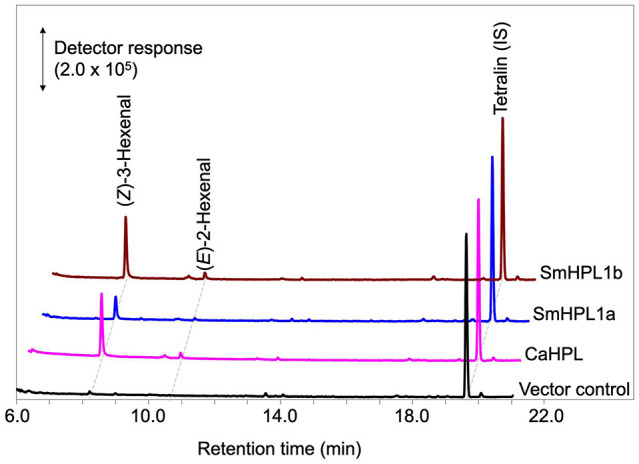
GC-MS analyses of the volatile products formed by recombinant SmHPL1a (blue) and SmHPL1b (brown) from 13HPOT. As the positive control, volatile products formed by bell pepper HPL (CaHPL) from 13HPOT are also shown (magenta). The vector control was run with the membrane fraction of *E. coli* cells harboring pET15b (black).

The open reading frame of SmCYP74L1 was obtained by PCR cloning of genomic DNA and sequenced. Two clones were retained, one had 100% similarity to the sequence reported in the genome database, while the other showed nine nucleotide substitutions resulting in the substitution of six amino acids (Supplementary Figure 5). We tentatively named the identical gene to that in the database as *SmCYP74L1a* and its allelic gene *SmCYP74L1b*. The membrane fraction prepared from *E. coli* lysate expressing recombinant proteins derived from the open reading frame of these two genes degraded 13HPOT *in vitro*. GC-MS analyses of the products obtained from the reaction of the membrane fraction harboring recombinant SmCYP74L1a or SmCYP74L1b protein with 13HPOT showed the formation of (*Z*)-3-hexenal as the main product (Figure 5). LC-MS/MS analyses of the non-volatile products indicated the formation of 12-oxo-(*Z*)-9-dodecenoic acid with both recombinant enzymes (Figure 4). No peaks corresponding to the products of AOS, DES, or EAS activities were detected. Accordingly, we retained the name SmHPL1a for SmCYP74L1a and SmHPL1b for SmCYP74L1b.

*Escherichia coli* membrane expressing recombinant SmHPL1b showed higher HPL activity (Figure 5); therefore, we further analyzed its properties. The highest activity was observed at pH 5.5, and under standard conditions with 40 μM of the four HPOs, recombinant SmHPL1b showed the greatest activity with 13HPOT (Table 1). In contrast, 13HPOD had only 6% activity with 13HPOT, whereas 9HPOT and 9HPOD were only slightly catalyzed by recombinant SmHPL1b. The recombinant enzyme followed Michaelis-Menten kinetics, and the *Km* value with 13HPOT was estimated to be 31.4 μM (Supplementary Figure 6).

### Gene Expression

**FIGURE 6:**
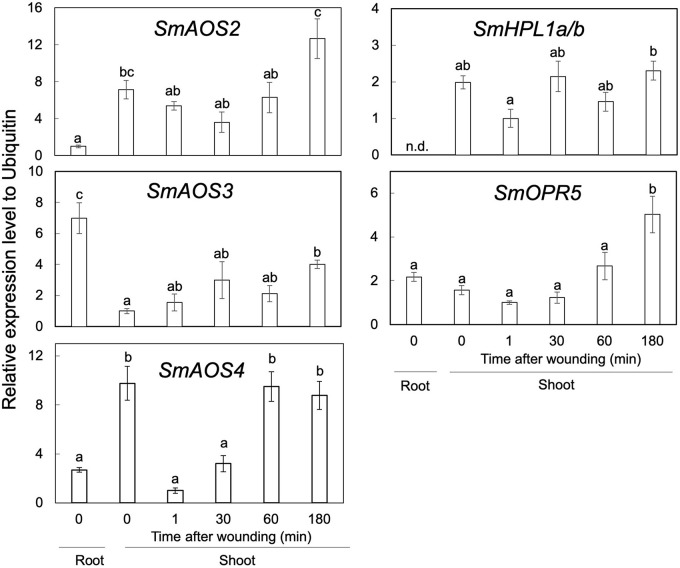
Gene-expression profiles of *SmAOS2*, *SmAOS3*, *SmAOS4*, *SmHPL1a/b*, and *SmOPR5* in the roots and the shoots. Those in the shoots after mechanical wounding are also shown. The expression data represent the mean ± standard error of four biological replicates. Different letters indicate significant difference (*P* < 0.05, one-way ANOVA, Tukey-Kramer). n.d., not detected.

RT-qPCR analyses showed that the transcription levels of *SmAOS2* and *SmAOS4* in the shoots were higher than those in the roots, while that of *SmAOS3* in the roots was higher than that in the shoots (Figure 6). The level of *SmAOS3* in the shoots slightly increased after mechanical wounding. The expression level of *SmAOS4* immediately dropped after mechanical wounding and slowly returned to the original level after 60 min of wounding. Expression of *SmHPL1a/b* was rather specific to the shoots and the expression in the roots was not detected. The expression level varied slightly after mechanical wounding on the shoots, but no obvious induction or suppression after wounding was observed. The transcription level of *SmOPR5* increased after mechanical wounding as reported previously (Pratiwi et al., 2017).

### Phylogenetic Tree

**FIGURE 7:**
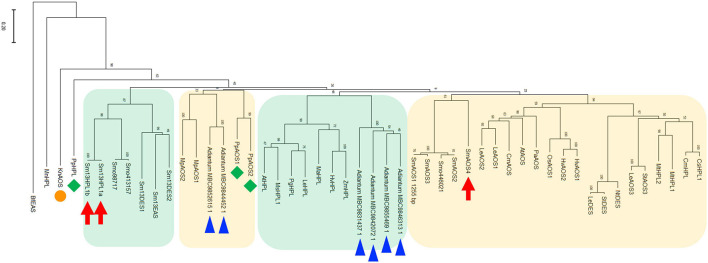
Phylogenetic analysis of SmHPL1a and b and SmAOS4 with the CYP74 enzymes from different species. Phylogenetic analysis was performed in MEGAX using the maximum likelihood method. The details of the sequences used here are shown in Supplementary Table 3. SmHPL1a/b and SmAOS4 are highlighted with red arrows. HPL and AOSs from *P. patens* are highlighted with green diamonds, and *K. nitens* AOS is with an orange circle. The genes found with transcriptome analyses on *A. capillus-veneris* are highlighted with blue triangles. The clade consisting of HPL, DES, and EAS from *S. moellendorffii*, and the one of seed plant HPLs are shown with green background, respectively. The clade mainly consisting of bryophyte AOSs, and the one of land plant AOSs, DESs, and 9/13HPLs are shown with orange background, respectively.

The phylogenetic tree with SmHPL1a, SmHPL1b, and SmAOS4, together with representatives of functionally characterized plant CYP74s and CYP74-like genes found with the transcriptome data of *Adiantum capillus-veneris* (Ni et al., 2020) showed that SmHPL1a and b were grouped with DES and EAS from *S. moellendorffii*, which consisted of a clade distinctive to the clade with seed plant HPLs. SmAOS4 is located in another clade closely related to the one consisting of angiosperm AOSs and 9/13HPLs (Figure 7). The six CYP74-like proteins found with the transcriptome data of *A. capillus-veneris* were divided into two groups, one in the angiosperm HPL clade and the other in the bryophyte AOS clade.

## Discussion

Based on the results presented in this study and those in previous reports (Hatanaka et al., 1978; Croisier et al., 2010), the ability of GLV-burst appears to have been acquired when the lycophyte species diverged from the plant lineage. GLVs are involved in the defense against pathogens and herbivores in angiosperms (Matsui, 2006; Ameye et al., 2018). The quick formation of GLVs at the damaged sites accounts for the efficient defensive effects through methods such as deterring actively feeding herbivores or attracting carnivores that prey on these herbivores. Cues strategically emitted from the appropriate place and time should provide reliable information for guiding carnivores to their prey. The fossil records indicate that lycophytes from the Permian to Triassic periods were attacked by various kinds of herbivores (Labandeira et al., 2016); thus, the plants at those geological ages would have employed several defensive strategies, including GLV-burst.

Interestingly, most bryophytes showed the ability to quickly form 1-octen-3-ol as opposed to GLVs after mechanical wounding of their thalli. This compound is a common volatile among fungi, and its involvement in the defense against fungivores, including arthropods and nematodes, is assumed (Inamdar et al., 2020). It is likely that this rapid formation of 1-octen-3-ol after mechanical wounding could also be part of the defense against bryophyte feeders, although direct evidence of this is lacking. Among vascular plants, the distribution of 1-octen-3-ol is limited, and it is found only in some angiosperms of the orders Fabales and Lamiales (Matsui et al., 2018; Lawson et al., 2021). Some lycophytes formed 1-octen-3-ol, but this ability appeared indistinct in vascular plants. In summary, the ability to form 1-octen-3-ol appeared to be replaced by GLV-burst when the lycophytes emerged from the plant lineage. Labeling experiments supported that C20 fatty acids, such as arachidonic acid and icosapentaenoic acid, were the predominant precursors for 1-octen-3-ol formation in bryophytes (Croisier et al., 2010). With a moss *Physcomitrella patens*, disruption of the *PpHPL* that was essential to formation of (*E*)-2-nonenal showed no effect on the ability to form 1-octen-3-ol (Stumpe et al., 2006). Therefore, PpHPL is not involved in 1-octen-3-ol formation, but the a multifunctional lipoxygenase catalyzes the oxygenation of arachidonic acid to 12-hydroperoxide as well as the subsequent cleavage reaction to form 1-octen-3-ol (Senger et al., 2005). Arachidonic acid and icosapentaenoic acid are common in bryophytes but are rare in vascular plants (Lu et al., 2019). Accordingly, the timing of the change from 1-octen-3-ol to GLVs coincides to a degree with the loss of C20 fatty acids, as well as with the period when the distribution of arachidonic acid began showing an inverse correlation with jasmonic acid (Gachet et al., 2017). The composition of oxylipins is modified in response to changes in the fatty acid composition during plant evolution. Accordingly, the enzyme systems that form oxylipin volatiles should be replaced from those related to 1-octen-3-ol to those involved in GLV-burst, justifying the acquisition of SmHPL1a and SmHPL1b as part of this process. The notable exception found with *Dicranum scoparium* that showed the substantial ability of GLV-burst should not be ignored. Another example of the exception was reported with *Neckera complanata* (Croisier et al., 2010). These are two species found among 29 species of bryophytes examined so far. *N. complanata* belongs to Hypnales and *D. scoparium* belongs to Dicranales, and they are distant from each other in the evolutionary tree. In the genome of the model bryophytes *M. polymorpha* and *P. pantens*, no gene encoding 13HPL has been found (Scholz et al., 2012; Koeduka et al., 2015). Taking together, it is likely that *D. scoparium* and *N. complanata* acquired GLV-burst capabilities due to convergent evolution. An example of the acquisition of metabolic capacity through convergent evolution was recently reported in a moss *Calohypnum plumiforme* (formerly *Hypnum plumaeforme*) (Hypnales) (Mao et al., 2020). This hypothesis should be examined as one of our next challenges.

**FIGURE 8:**
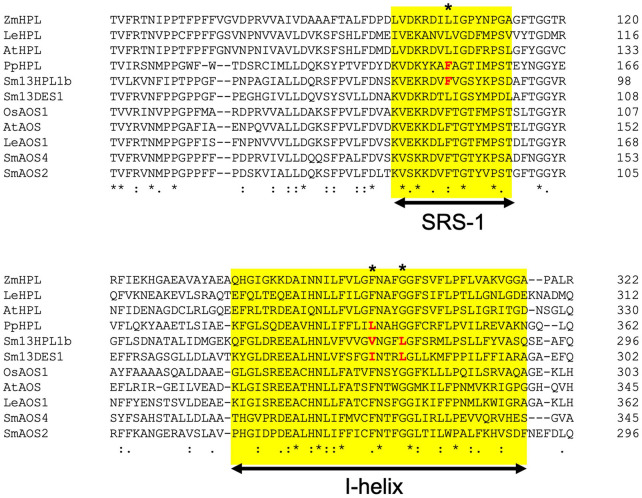
Multialignment of representative seed plant HPLs and AOSs with SmHPL1b, SmDES1, SmAOS4, and SmAOS2. The substrate recognition site (SRS)-1 and I-helix are highlighted with yellow background. The amino acids specific to non-seed CYP74s are shown in red. The position of F/L toggle is shown with asterisk.

*Klebsormidium nitens* has only one CYP74 gene in its genome and it encodes 13AOS (Koeduka et al., 2015). BLASTP analysis with genome sequences of the nine species belonging to Chlorophytes available in Phytozome 13 yielded no genes that were significantly similar to either *K. nitens* AOS or SmHPL1a/b. It has been reported that *Spirogloea muscicola* gen. nov., belonging to subaerial Zygnematophyceae, diversified after Klebsormidium, has one gene related to AOS in its genome (Cheng et al., 2019); therefore, it is suggested that *K. nitens* AOS is likely the closest to the common ancestor of the CYP74 genes that are widely found in extant terrestrial plants (Figure 7). In the moss *P. patens*, PpHPL that has the HPL activity moderately specific to linoleic acid 9-hydroperoxide (Stumpe et al., 2006) was first acquired from the ancestral CYP74 gene. *S. moellendorffii* likely adopted the CYP74 gene related to PpHPL that was further diversified into 13HPL, DES, and EAS. Another diversification of PpHPL-related ancestral gene resulted in three clades consisting of bryophyte AOS, angiosperm 13HPL, and vascular plant AOS/DES/HPL (Figure 7). Unexpectedly, genes found with a monilophyte *Adiantum capillus-veneris* locate in the clade of bryophyte AOS and that of vascular plant AOS/DES/HPL. Based on these results, it is suggested that 13HPL might have been acquired independently in *S. moellendorffii* and angiosperms. In fact, SmHPL1a/b does not follow the “F/L toggle rule” exclusively conserved among angiosperm HPL and AOS (Lee et al., 2008; Scholz et al., 2012; Toporkova et al., 2019; Figure 8). The structural analysis unambiguously indicated that the Phe residue located in the active site of AtAOS stabilized an intermediary-formed carbon-centered radical that led to allene oxide, and Leu at the same position led to hemiacetal that finally caused the formation of HPL products (Lee et al., 2008). SmHPL1a/b are the exception among HPLs that have Phe at the toggle in the substrate recognition site (SRS)-1 domain (Figure 8), and other than SmHPL1a/b, only PpHPL contains Phe at the toggle. Amino acid replacements unique to PpHPL, SmHPL1a/b, or SmDES1 are also found in the I-helix, which is referred to as the oxygen-binding domain (Figure 8). Accordingly, it is assumed that the structural determinants strictly followed by HPL and AOS in angiosperms are not applicable to those of bryophytes and lycophytes, which supports the hypothesis that HPL genes were independently acquired in *S. moellendorffii* and angiosperms.

Overall, all CYP74s in the plant lineage could be derived from a common ancestral gene close to *K. nitens* AOS. CYP74 is characterized as the P450 that lacks monooxygenase activity, and instead has the ability to rearrange fatty acid hydroperoxides through the homolytic scission of the hydroperoxyl group (Brash, 2009). All enzymes belonging to CYP74s share the first part of the reaction, that is, the homolytic scission of the hydroperoxyl group to form epoxyallylic radicals. The fate of the reactive carbon-centered radical intermediate is the determinant of the products, which confirms whether the enzyme of each CYP74 is denoted as HPL or AOS. The fate is likely determined by a few amino acid residues located at the active site (Lee et al., 2008; Scholz et al., 2012; Toporkova et al., 2019). Therefore, site-directed mutagenesis of a few amino acid residues at the active site allowed the interconversion of HPL to AOS and HPL/EAS to AOS (Lee et al., 2008; Scholz et al., 2012; Toporkova et al., 2019). This characteristic feature of CYP74s shows that HPL could have developed independently in *S. moellendorffii* and angiosperms, allowing for the diversification of CYP74 enzymes, with the interconversion of their catalytic activities. It is also possible that 13HPL was acquired independently in *N. complanata* and *D. scoparium*. Further collection of 13HPL genes in non-seed plants should be conducted to elucidate the structural basis of how the development of 13HPL in lycophytes and monilophytes proceeded.

This hypothesis, in turn, indicates that the GLV-burst plays a significant role in improving plant fitness during evolution after the loss of the ability to form 1-octen-3-ol. The advantageous effects of the GLV-burst have been well documented in angiosperms to date (Matsui, 2006; Ameye et al., 2018). In lycophytes, this ability is expected to increase their fitness, but further studies are needed to determine the benefits to this group. The ability of the GLV-burst in a few bryophytes found in this study is substantial, and it is expected that these bryophyte species employed convergent evolution to convert CYP74s encoding 9HPL or AOS into 13HPL to benefit from the GLV-burst. This is the hypothesis that requires further study.

## Data Availability Statement

The datasets presented in this study can be found in online repositories. The names of the repository/repositories and accession number(s) can be found in the article/Supplementary Material.

## Author Contributions

KM and MT participated in the design of the experiment. MT performed the majority of the experiments. KM, MT, and TK wrote the manuscript. All authors contributed to the article and approved the submitted version.

## Conflict of Interest

The authors declare that the research was conducted in the absence of any commercial or financial relationships that could be construed as a potential conflict of interest.

## Publisher’s Note

All claims expressed in this article are solely those of the authors and do not necessarily represent those of their affiliated organizations, or those of the publisher, the editors and the reviewers. Any product that may be evaluated in this article, or claim that may be made by its manufacturer, is not guaranteed or endorsed by the publisher.

## Footnotes

1
https://phytozome.jgi.doe.gov/

2
phytozome.jgi.doe.gov/pz/portal.html

3
https://conekt.sbs.ntu.edu.sg/
