# Supplementary material for: Green Leaf Volatile-Burst in Selaginella moellendorffii
Source: Front Plant Sci. 2021 Oct 27;12:731694. doi: 10.3389/fpls.2021.731694 (PMC8578206; doi:10.3389/fpls.2021.731694)
Supplement: Supplementary file 2 [file Presentation_1.PPTX]

## Slide 1
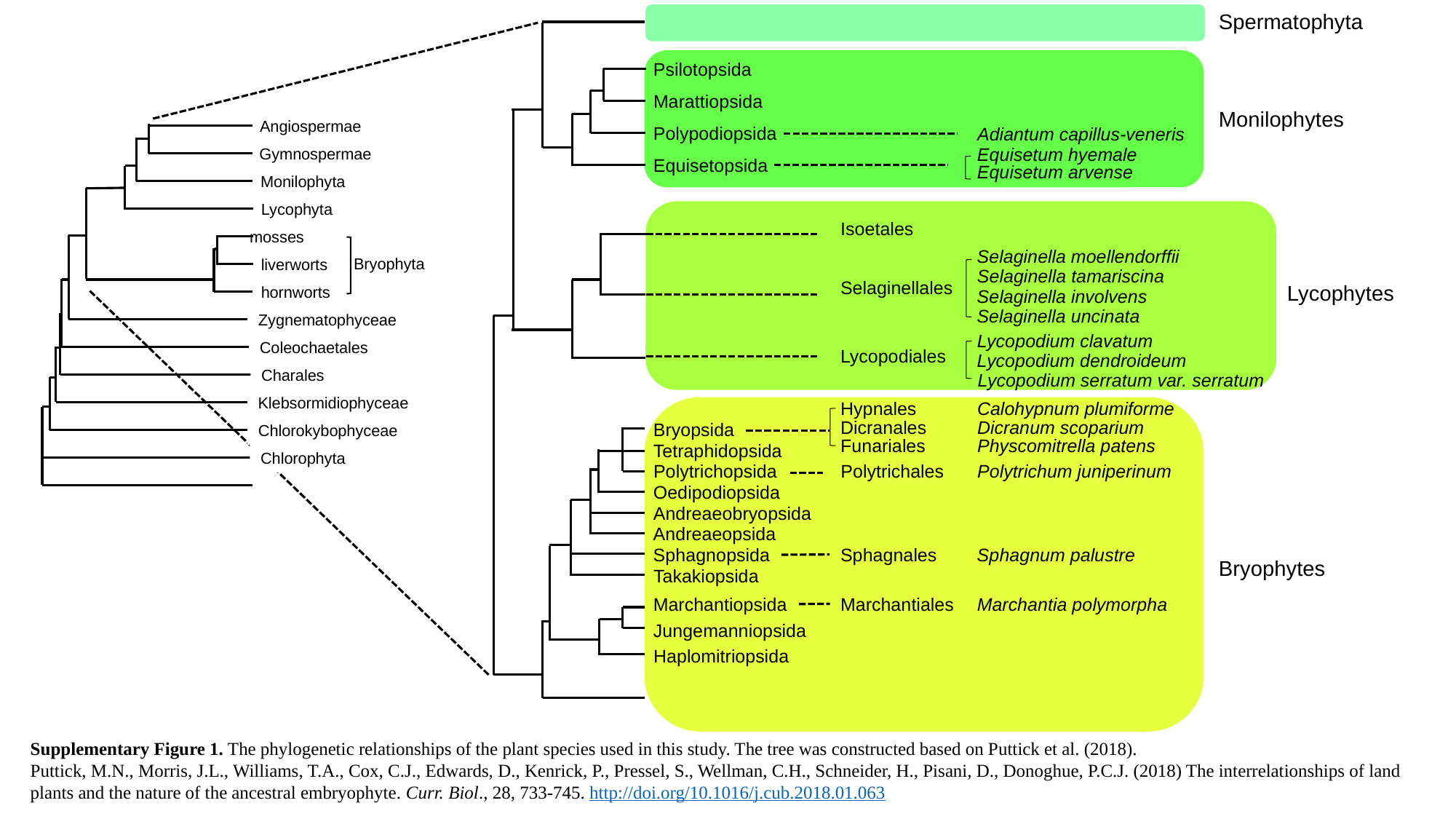

Spermatophyta
Psilotopsida
Marattiopsida
Monilophytes
Angiospermae
Polypodiopsida
Adiantum capillus-veneris
Equisetum hyemale
Gymnospermae
Equisetopsida
Equisetum arvense
Monilophyta
Lycophyta
Isoetales
mosses
Selaginella moellendorffii
Bryophyta
liverworts
Selaginella tamariscina
Selaginellales
Lycophytes
hornworts
Selaginella involvens
Selaginella uncinata
Zygnematophyceae
Lycopodium clavatum
Coleochaetales
Lycopodiales
Lycopodium dendroideum
Charales
Lycopodium serratum var. serratum
Klebsormidiophyceae
Hypnales
Calohypnum plumiforme
Dicranales
Dicranum scoparium
Bryopsida
Chlorokybophyceae
Funariales
Physcomitrella patens
Tetraphidopsida
Chlorophyta
Polytrichopsida
Polytrichales
Polytrichum juniperinum
Oedipodiopsida
Andreaeobryopsida
Andreaeopsida
Sphagnopsida
Sphagnales
Sphagnum palustre
Bryophytes
Takakiopsida
Marchantiopsida
Marchantiales
Marchantia polymorpha
Jungemanniopsida
Haplomitriopsida
Supplementary Figure 1. The phylogenetic relationships of the plant species used in this study. The tree was constructed based on Puttick et al. (2018).
Puttick, M.N., Morris, J.L., Williams, T.A., Cox, C.J., Edwards, D., Kenrick, P., Pressel, S., Wellman, C.H., Schneider, H., Pisani, D., Donoghue, P.C.J. (2018) The interrelationships of land plants and the nature of the ancestral embryophyte. Curr. Biol., 28, 733-745. http://doi.org/10.1016/j.cub.2018.01.063

## Slide 2
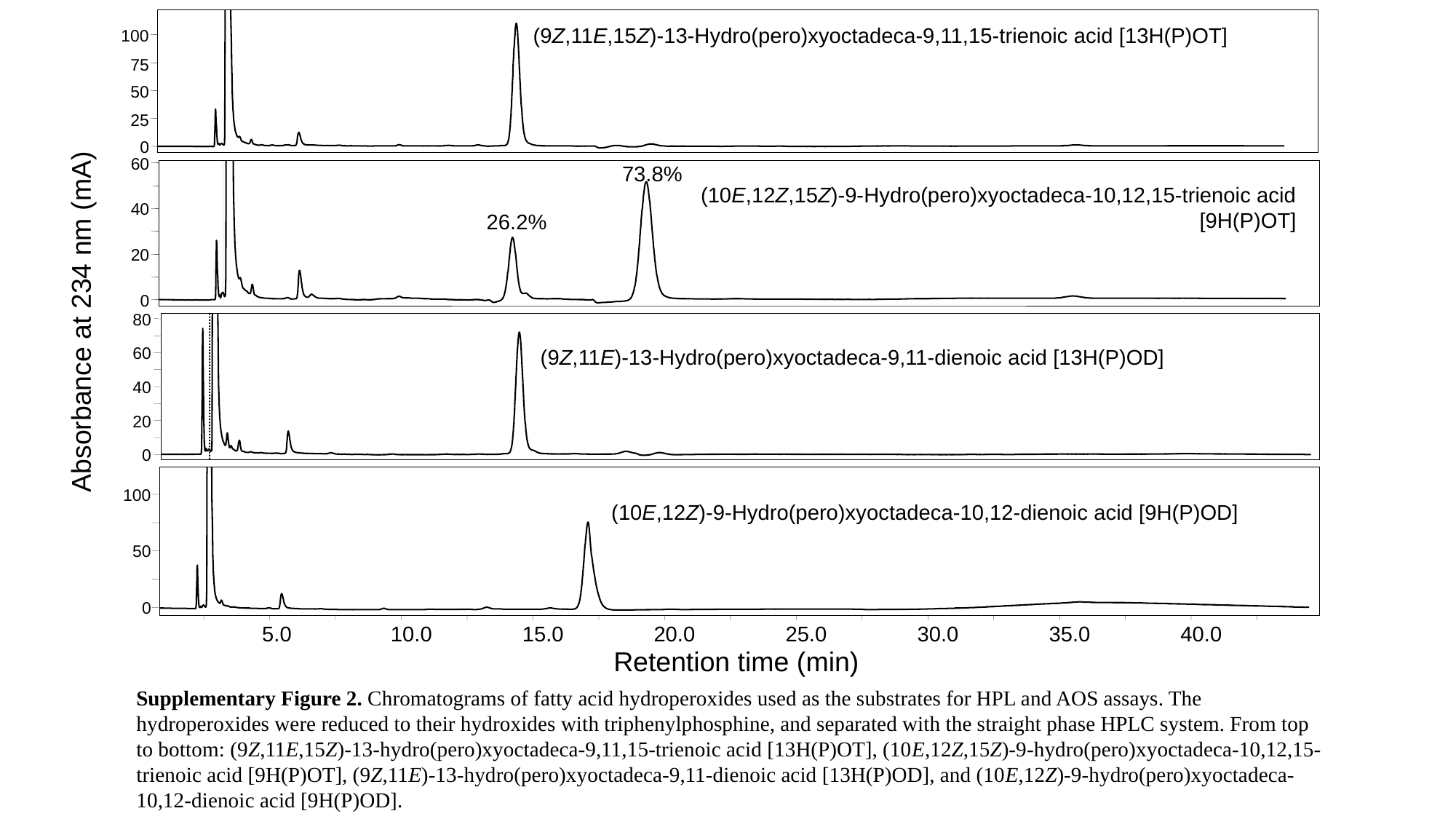

(9Z,11E,15Z)-13-Hydro(pero)xyoctadeca-9,11,15-trienoic acid [13H(P)OT]
100
75
50
25
0
60
73.8%
(10E,12Z,15Z)-9-Hydro(pero)xyoctadeca-10,12,15-trienoic acid
 [9H(P)OT]
40
26.2%
20
0
Absorbance at 234 nm (mA)
80
60
(9Z,11E)-13-Hydro(pero)xyoctadeca-9,11-dienoic acid [13H(P)OD]
40
20
0
100
(10E,12Z)-9-Hydro(pero)xyoctadeca-10,12-dienoic acid [9H(P)OD]
50
0
5.0
10.0
15.0
20.0
25.0
30.0
35.0
40.0
Retention time (min)
Supplementary Figure 2. Chromatograms of fatty acid hydroperoxides used as the substrates for HPL and AOS assays. The hydroperoxides were reduced to their hydroxides with triphenylphosphine, and separated with the straight phase HPLC system. From top to bottom: (9Z,11E,15Z)-13-hydro(pero)xyoctadeca-9,11,15-trienoic acid [13H(P)OT], (10E,12Z,15Z)-9-hydro(pero)xyoctadeca-10,12,15-trienoic acid [9H(P)OT], (9Z,11E)-13-hydro(pero)xyoctadeca-9,11-dienoic acid [13H(P)OD], and (10E,12Z)-9-hydro(pero)xyoctadeca-10,12-dienoic acid [9H(P)OD].

## Slide 3
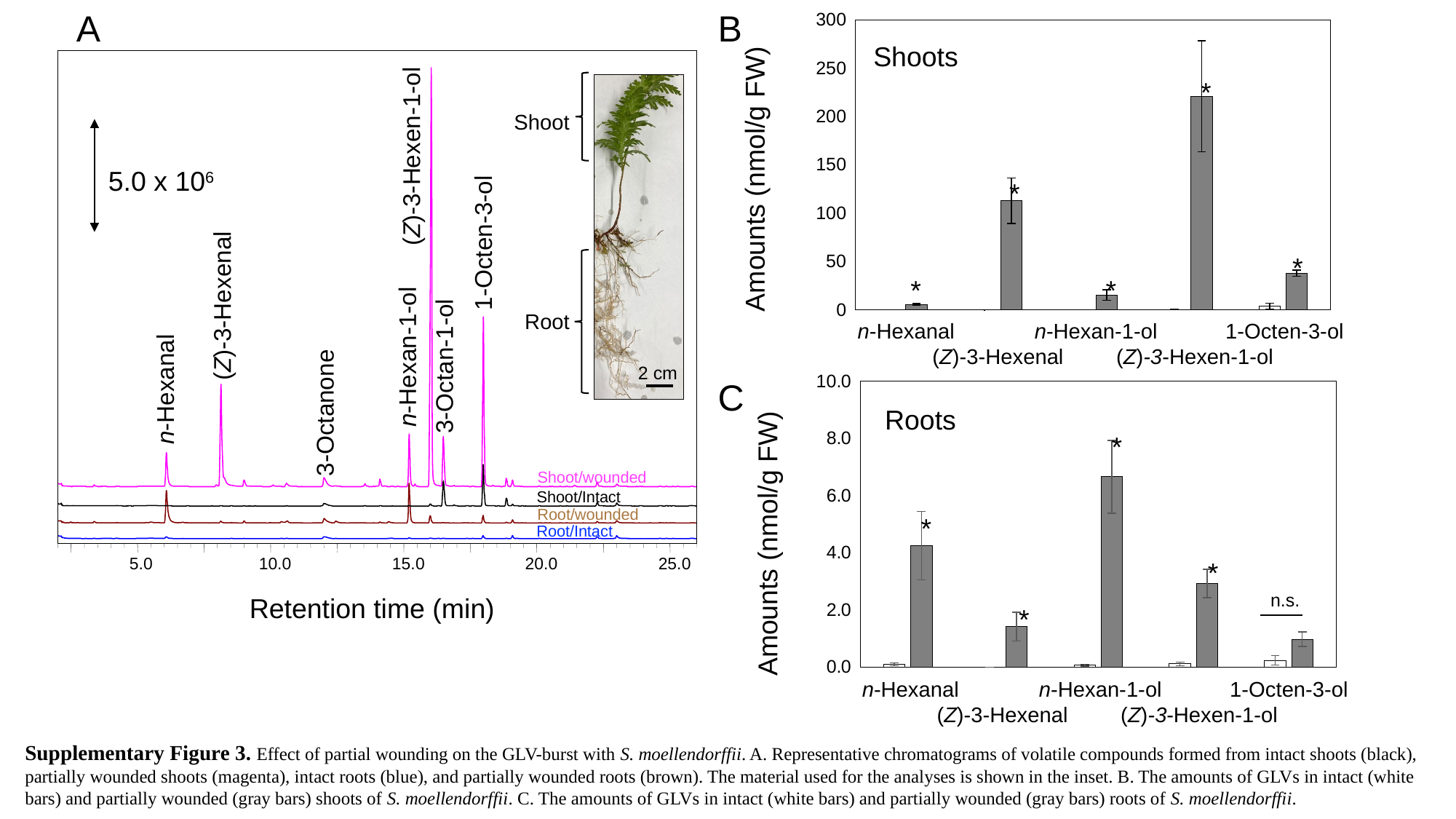

A
B
### Chart
| Category | Int avg | Cut avg |
|---|---|---|
| Hexanal | 0.009365186925247198 | 5.406038592098294 |
| 3-Hexenal | 0.036533533524610286 | 112.74159311495445 |
| n-Hexanol | 0.02155234462343854 | 15.386461970862676 |
| 3-Hexen-1-ol | 0.4144879063467091 | 220.86048227108145 |
| 1-Octen-3-ol | 3.611429900987116 | 37.927808218407726 |Shoots
*
Shoot
(Z)-3-Hexen-1-ol
Amounts (nmol/g FW)
5.0 x 10^6^
*
1-Octen-3-ol
*
*
*
(Z)-3-Hexenal
Root
n-Hexanal
n-Hexan-1-ol
1-Octen-3-ol
n-Hexan-1-ol
(Z)-3-Hexenal
(Z)-3-Hexen-1-ol
3-Octan-1-ol
2 cm
### Chart
| Category | Int avg | Cut avg |
|---|---|---|
| Hexanal | 0.09388688584256276 | 4.245215083602669 |
| 3-Hexenal | 0.0 | 1.4124758409027152 |
| n-Hexanol | 0.0689506917992666 | 6.657682050602176 |
| 3-Hexen-1-ol | 0.11264087378551145 | 2.9186909466213926 |
| 1-Octen-3-ol | 0.2328259633028472 | 0.971625081409897 |n-Hexanal
C
3-Octanone
Roots
*
Shoot/wounded
Shoot/Intact
Root/wounded
*
Root/Intact
Amounts (nmol/g FW)
*
5.0
10.0
15.0
20.0
25.0
n.s.
Retention time (min)
*
n-Hexanal
n-Hexan-1-ol
1-Octen-3-ol
(Z)-3-Hexenal
(Z)-3-Hexen-1-ol
Supplementary Figure 3. Effect of partial wounding on the GLV-burst with S. moellendorffii. A. Representative chromatograms of volatile compounds formed from intact shoots (black), partially wounded shoots (magenta), intact roots (blue), and partially wounded roots (brown). The material used for the analyses is shown in the inset. B. The amounts of GLVs in intact (white bars) and partially wounded (gray bars) shoots of S. moellendorffii. C. The amounts of GLVs in intact (white bars) and partially wounded (gray bars) roots of S. moellendorffii.

## Slide 4
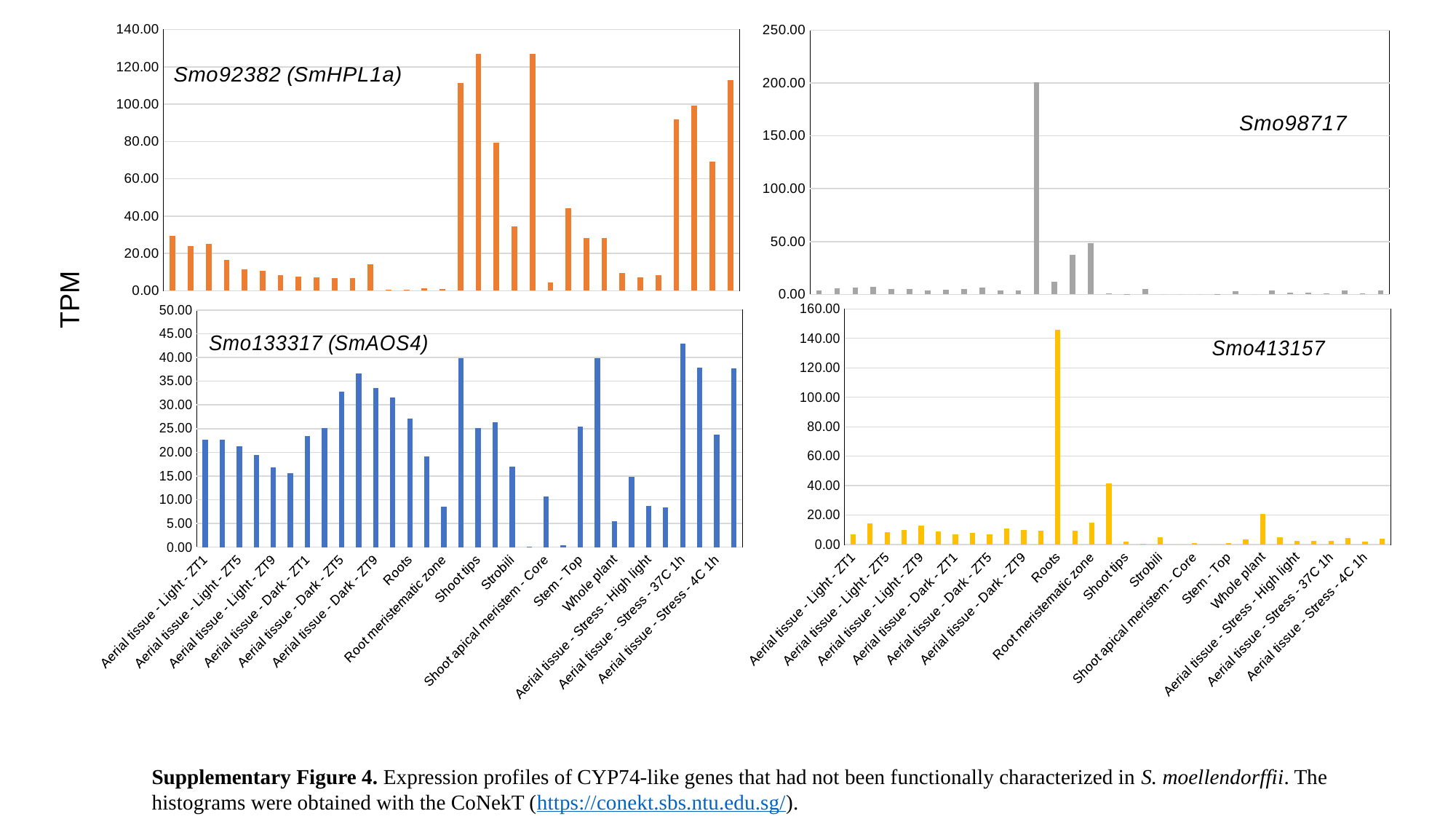

### Chart:
| Category | Smo98717 |
|---|---|
| Aerial tissue - Light - ZT1 | 3.85938183333333 |
| Aerial tissue - Light - ZT3 | 5.65526666666666 |
| Aerial tissue - Light - ZT5 | 6.66578 |
| Aerial tissue - Light - ZT7 | 6.88056 |
| Aerial tissue - Light - ZT9 | 4.82912666666666 |
| Aerial tissue - Light - ZT11 | 4.79895333333333 |
| Aerial tissue - Dark - ZT1 | 3.95160333333333 |
| Aerial tissue - Dark - ZT3 | 4.28185333333333 |
| Aerial tissue - Dark - ZT5 | 5.34952 |
| Aerial tissue - Dark - ZT7 | 6.22987 |
| Aerial tissue - Dark - ZT9 | 3.96837666666666 |
| Aerial tissue - Dark - ZT11 | 3.7482 |
| Roots | 200.9775 |
| Root elongation and differentiation zone | 11.7252666666666 |
| Root meristematic zone | 37.6858333333333 |
| Aerial roots and rhizophores | 48.5495499999999 |
| Shoot tips | 0.73595 |
| Microphyll | 0.1261805 |
| Strobili | 4.83577 |
| Shoot apical meristem - AC | 0.0 |
| Shoot apical meristem - Core | 0.0 |
| Shoot apical meristem - P1 | 0.0 |
| Stem - Top | 0.627393499999999 |
| Stem - Bottom | 3.268755 |
| Whole plant | 0.0 |
| Aerial tissue - Stress - Dark | 3.75758499999999 |
| Aerial tissue - Stress - High light | 1.508005 |
| Aerial tissue - Stress - Cont | 1.93215 |
| Aerial tissue - Stress - 37C 1h | 1.295092 |
| Aerial tissue - Stress - 37C 3h | 3.75145 |
| Aerial tissue - Stress - 4C 1h | 0.9952595 |
| Aerial tissue - Stress - 4C 3h | 3.9503775 |
### Chart:
| Category | Smo92382 (SmHPL1a) |
|---|---|
| Aerial tissue - Light - ZT1 | 29.3095666666666 |
| Aerial tissue - Light - ZT3 | 23.9907333333333 |
| Aerial tissue - Light - ZT5 | 24.9632666666666 |
| Aerial tissue - Light - ZT7 | 16.5852666666666 |
| Aerial tissue - Light - ZT9 | 11.5344766666666 |
| Aerial tissue - Light - ZT11 | 10.5254466666666 |
| Aerial tissue - Dark - ZT1 | 8.39960333333333 |
| Aerial tissue - Dark - ZT3 | 7.41921666666666 |
| Aerial tissue - Dark - ZT5 | 7.06634 |
| Aerial tissue - Dark - ZT7 | 6.89418 |
| Aerial tissue - Dark - ZT9 | 6.65053666666666 |
| Aerial tissue - Dark - ZT11 | 14.0162666666666 |
| Roots | 0.4340725 |
| Root elongation and differentiation zone | 0.517287333333333 |
| Root meristematic zone | 1.37040333333333 |
| Aerial roots and rhizophores | 1.044681 |
| Shoot tips | 111.530149999999 |
| Microphyll | 126.92835 |
| Strobili | 79.40495 |
| Shoot apical meristem - AC | 34.5415333333333 |
| Shoot apical meristem - Core | 127.114 |
| Shoot apical meristem - P1 | 4.60742 |
| Stem - Top | 44.4003 |
| Stem - Bottom | 28.10625 |
| Whole plant | 28.1239 |
| Aerial tissue - Stress - Dark | 9.41974 |
| Aerial tissue - Stress - High light | 7.329825 |
| Aerial tissue - Stress - Cont | 8.49744 |
| Aerial tissue - Stress - 37C 1h | 91.9958 |
| Aerial tissue - Stress - 37C 3h | 99.1012 |
| Aerial tissue - Stress - 4C 1h | 69.09635 |
| Aerial tissue - Stress - 4C 3h | 113.0575 |
### Chart:
| Category | Smo413157 |
|---|---|
| Aerial tissue - Light - ZT1 | 6.87404833333333 |
| Aerial tissue - Light - ZT3 | 14.2131333333333 |
| Aerial tissue - Light - ZT5 | 8.23771666666666 |
| Aerial tissue - Light - ZT7 | 9.90007 |
| Aerial tissue - Light - ZT9 | 12.8707333333333 |
| Aerial tissue - Light - ZT11 | 8.86456333333333 |
| Aerial tissue - Dark - ZT1 | 7.20255666666666 |
| Aerial tissue - Dark - ZT3 | 7.72786666666666 |
| Aerial tissue - Dark - ZT5 | 6.75863 |
| Aerial tissue - Dark - ZT7 | 11.0500966666666 |
| Aerial tissue - Dark - ZT9 | 9.68856666666666 |
| Aerial tissue - Dark - ZT11 | 9.40364666666666 |
| Roots | 145.7345 |
| Root elongation and differentiation zone | 9.57254333333333 |
| Root meristematic zone | 14.96683 |
| Aerial roots and rhizophores | 41.5638 |
| Shoot tips | 2.10005 |
| Microphyll | 0.697037 |
| Strobili | 5.08421 |
| Shoot apical meristem - AC | 0.0 |
| Shoot apical meristem - Core | 0.827265 |
| Shoot apical meristem - P1 | 0.0 |
| Stem - Top | 1.159943 |
| Stem - Bottom | 3.491855 |
| Whole plant | 20.7302499999999 |
| Aerial tissue - Stress - Dark | 4.85314999999999 |
| Aerial tissue - Stress - High light | 2.73335 |
| Aerial tissue - Stress - Cont | 2.73045 |
| Aerial tissue - Stress - 37C 1h | 2.39658999999999 |
| Aerial tissue - Stress - 37C 3h | 4.353725 |
| Aerial tissue - Stress - 4C 1h | 1.78223999999999 |
| Aerial tissue - Stress - 4C 3h | 3.735225 |TPM
### Chart:
| Category | Smo133317 (SmAOS4) |
|---|---|
| Aerial tissue - Light - ZT1 | 22.70965 |
| Aerial tissue - Light - ZT3 | 22.6693666666666 |
| Aerial tissue - Light - ZT5 | 21.3377666666666 |
| Aerial tissue - Light - ZT7 | 19.4696 |
| Aerial tissue - Light - ZT9 | 16.8770666666666 |
| Aerial tissue - Light - ZT11 | 15.6161333333333 |
| Aerial tissue - Dark - ZT1 | 23.5067666666666 |
| Aerial tissue - Dark - ZT3 | 25.1779333333333 |
| Aerial tissue - Dark - ZT5 | 32.7932 |
| Aerial tissue - Dark - ZT7 | 36.5479666666666 |
| Aerial tissue - Dark - ZT9 | 33.6196 |
| Aerial tissue - Dark - ZT11 | 31.5476333333333 |
| Roots | 27.14725 |
| Root elongation and differentiation zone | 19.1379666666666 |
| Root meristematic zone | 8.48691 |
| Aerial roots and rhizophores | 39.8011 |
| Shoot tips | 25.0853 |
| Microphyll | 26.36855 |
| Strobili | 17.00975 |
| Shoot apical meristem - AC | 0.0875259666666666 |
| Shoot apical meristem - Core | 10.672075 |
| Shoot apical meristem - P1 | 0.489137999999999 |
| Stem - Top | 25.4876 |
| Stem - Bottom | 39.8051 |
| Whole plant | 5.539835 |
| Aerial tissue - Stress - Dark | 14.8652 |
| Aerial tissue - Stress - High light | 8.68325499999999 |
| Aerial tissue - Stress - Cont | 8.34988 |
| Aerial tissue - Stress - 37C 1h | 42.9153 |
| Aerial tissue - Stress - 37C 3h | 37.9025 |
| Aerial tissue - Stress - 4C 1h | 23.71815 |
| Aerial tissue - Stress - 4C 3h | 37.77375 |Supplementary Figure 4. Expression profiles of CYP74-like genes that had not been functionally characterized in S. moellendorffii. The histograms were obtained with the CoNekT (https://conekt.sbs.ntu.edu.sg/).

## Slide 5
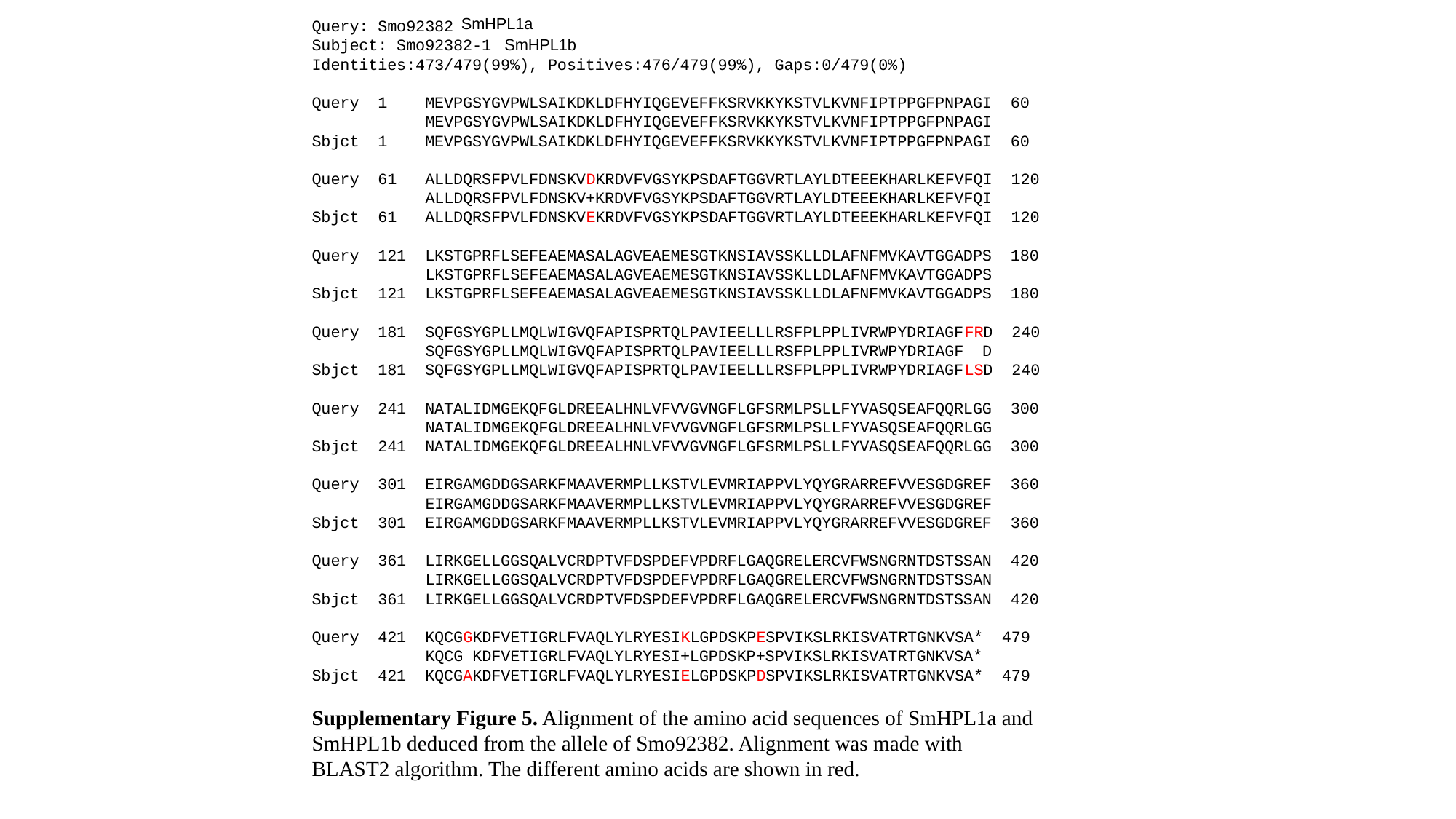

SmHPL1a
Query: Smo92382
Subject: Smo92382-1
Identities:473/479(99%), Positives:476/479(99%), Gaps:0/479(0%)
Query 1 MEVPGSYGVPWLSAIKDKLDFHYIQGEVEFFKSRVKKYKSTVLKVNFIPTPPGFPNPAGI 60
 MEVPGSYGVPWLSAIKDKLDFHYIQGEVEFFKSRVKKYKSTVLKVNFIPTPPGFPNPAGI
Sbjct 1 MEVPGSYGVPWLSAIKDKLDFHYIQGEVEFFKSRVKKYKSTVLKVNFIPTPPGFPNPAGI 60
Query 61 ALLDQRSFPVLFDNSKVDKRDVFVGSYKPSDAFTGGVRTLAYLDTEEEKHARLKEFVFQI 120
 ALLDQRSFPVLFDNSKV+KRDVFVGSYKPSDAFTGGVRTLAYLDTEEEKHARLKEFVFQI
Sbjct 61 ALLDQRSFPVLFDNSKVEKRDVFVGSYKPSDAFTGGVRTLAYLDTEEEKHARLKEFVFQI 120
Query 121 LKSTGPRFLSEFEAEMASALAGVEAEMESGTKNSIAVSSKLLDLAFNFMVKAVTGGADPS 180
 LKSTGPRFLSEFEAEMASALAGVEAEMESGTKNSIAVSSKLLDLAFNFMVKAVTGGADPS
Sbjct 121 LKSTGPRFLSEFEAEMASALAGVEAEMESGTKNSIAVSSKLLDLAFNFMVKAVTGGADPS 180
Query 181 SQFGSYGPLLMQLWIGVQFAPISPRTQLPAVIEELLLRSFPLPPLIVRWPYDRIAGFFRD 240
 SQFGSYGPLLMQLWIGVQFAPISPRTQLPAVIEELLLRSFPLPPLIVRWPYDRIAGF D
Sbjct 181 SQFGSYGPLLMQLWIGVQFAPISPRTQLPAVIEELLLRSFPLPPLIVRWPYDRIAGFLSD 240
Query 241 NATALIDMGEKQFGLDREEALHNLVFVVGVNGFLGFSRMLPSLLFYVASQSEAFQQRLGG 300
 NATALIDMGEKQFGLDREEALHNLVFVVGVNGFLGFSRMLPSLLFYVASQSEAFQQRLGG
Sbjct 241 NATALIDMGEKQFGLDREEALHNLVFVVGVNGFLGFSRMLPSLLFYVASQSEAFQQRLGG 300
Query 301 EIRGAMGDDGSARKFMAAVERMPLLKSTVLEVMRIAPPVLYQYGRARREFVVESGDGREF 360
 EIRGAMGDDGSARKFMAAVERMPLLKSTVLEVMRIAPPVLYQYGRARREFVVESGDGREF
Sbjct 301 EIRGAMGDDGSARKFMAAVERMPLLKSTVLEVMRIAPPVLYQYGRARREFVVESGDGREF 360
Query 361 LIRKGELLGGSQALVCRDPTVFDSPDEFVPDRFLGAQGRELERCVFWSNGRNTDSTSSAN 420
 LIRKGELLGGSQALVCRDPTVFDSPDEFVPDRFLGAQGRELERCVFWSNGRNTDSTSSAN
Sbjct 361 LIRKGELLGGSQALVCRDPTVFDSPDEFVPDRFLGAQGRELERCVFWSNGRNTDSTSSAN 420
Query 421 KQCGGKDFVETIGRLFVAQLYLRYESIKLGPDSKPESPVIKSLRKISVATRTGNKVSA* 479
 KQCG KDFVETIGRLFVAQLYLRYESI+LGPDSKP+SPVIKSLRKISVATRTGNKVSA*
Sbjct 421 KQCGAKDFVETIGRLFVAQLYLRYESIELGPDSKPDSPVIKSLRKISVATRTGNKVSA* 479
SmHPL1b
Supplementary Figure 5. Alignment of the amino acid sequences of SmHPL1a and SmHPL1b deduced from the allele of Smo92382. Alignment was made with BLAST2 algorithm. The different amino acids are shown in red.

## Slide 6
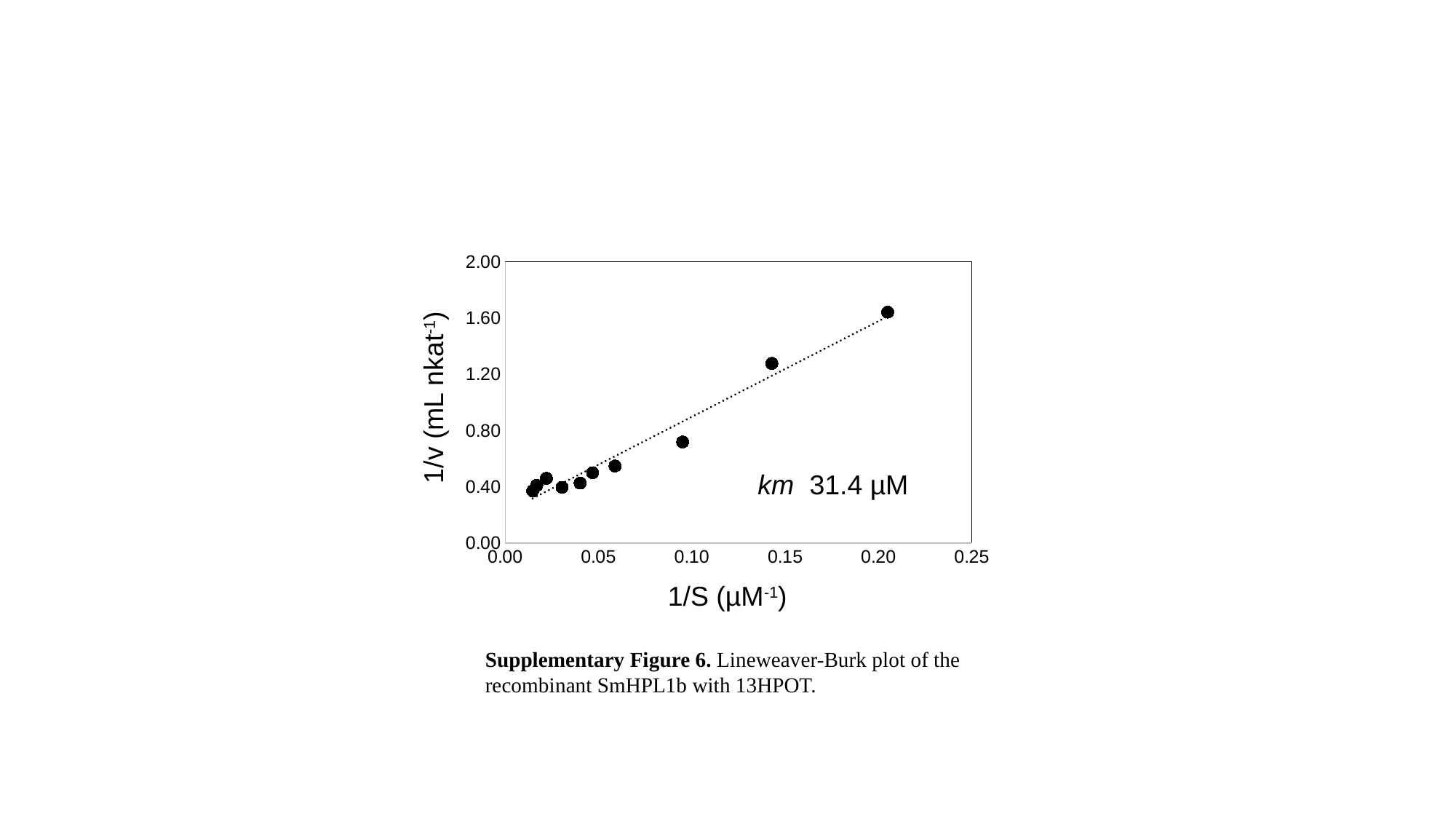

### Chart
| Category | 1/v (mL/nkat) |
|---|---|1/v (mL nkat-1)
km 31.4 µM
1/S (µM-1)
Supplementary Figure 6. Lineweaver-Burk plot of the recombinant SmHPL1b with 13HPOT.
