# Supplementary material for: Green Leaf Volatile-Burst in Selaginella moellendorffii
Source: Front Plant Sci. 2021 Oct 27;12:731694. doi: 10.3389/fpls.2021.731694 (PMC8578206; doi:10.3389/fpls.2021.731694)
Supplement: Supplementary file 3 [file Table_1.DOCX]

**Supplementary Table 1. The list of where to get the plants.**

| Plant Name | How to obtain |
| --- | --- |
| *Adiantum capillus-veneris* | Purchased from garden center "DAIKI" |
| *Equisetum hyemale* | Purchased from online store "Charm" |
| *Equisetum arvense* | Harvested at Yamaguchi University (2020/7/8) |
| *Selaginella moellendorffii* | Provided by Prof. Dr. Xie Xiaonan, Utsunomiya University, Japan |
| *Selaginella tamariscina* | Purchased from online store "Rakuten-Ichiba" |
| *Selaginella involvens* | Purchased from garden center "DAIKI" |
| *Selaginella uncinata* | Purchased from online store "Charm" |
| *Lycopodium clavatum* | Purchased from online store "Kita-no-sansai" |
| *Lycopodium dendroideum* | Purchased from online store "Charm" |
| *Lycopodium serratum var. serratum* | Purchased from online store "Charm" |
| *Calohypnum plumiforme*  (formerly *Hypnum plumaeforme*) | Purchased from online store "Kokerian" |
| *Dicranum scoparium* | Purchased from online store "Charm" |
| *Physcomitrella patens* | Provided by Prof. Dr. Mitsuyasu Hasebe, The National Institute for Basic Biology (NIBB), Japan |
| *Polytrichum juniperinum* | Harvested at Yamaguchi University (Sep 14, 2020) |
| *Sphagnum palustre* | Purchased from online store "Kokerian" |
| *Marchantia polymorpha* | Takaragaike-1 strain provided by Prof. Dr. Takayuki Kochi, Kyoto University, Japan |
|  |  |
| Online store "Charm" | <http://www.shopping-charm.jp/> |
| Online store "Rakuten-ichiba" | <https://www.ranking.rakuten.co.jp/> |
| Online store "Kita-no-sansai" | [https://kitanosansai.ocnk.net](https://kitanosansai.ocnk.net/) |
| Online store "Kokerian" | <https://www.rakuten.co.jp/koke-rian/> |
