# Supplementary material for: Green Leaf Volatile-Burst in Selaginella moellendorffii
Source: Front Plant Sci. 2021 Oct 27;12:731694. doi: 10.3389/fpls.2021.731694 (PMC8578206; doi:10.3389/fpls.2021.731694)
Supplement: Supplementary file 4 [file Table_2.DOCX]

**Supplementary Table 2. Primers used in this study.**

| Primer Name | Sequence 5' to 3' | Target gene | Purpose | Reference |
| --- | --- | --- | --- | --- |
| SmHPL1_F | CACCATGGAGGTCCCCGGAAGCTATG | [SmHPL (XP_002969700.1)](https://www.ncbi.nlm.nih.gov/protein/XP_002969700.1) | Topo cloning, Sequencing | This study |
| SmHPL1_R | CTAGGCACTTACTTTGTTGCCAG | [SmHPL (XP_002969700.1)](https://www.ncbi.nlm.nih.gov/protein/XP_002969700.1) | Topo cloning, Sequencing | This study |
| SmHPL1_Seq1F | GCCCGTCTCAAGGAATTCGTG | [SmHPL (XP_002969700.2)](https://www.ncbi.nlm.nih.gov/protein/XP_002969700.1) | Sequencing | This study |
| SmHPL1_Seq1R | CTCCAGCACGGTCGATTTGAG | [SmHPL (XP_002969700.3)](https://www.ncbi.nlm.nih.gov/protein/XP_002969700.1) | Sequencing | This study |
| SmHPL1_Seq2F | AAGTTCATGGCCGCGGTG | [SmHPL (XP_002969700.4)](https://www.ncbi.nlm.nih.gov/protein/XP_002969700.1) | Sequencing | This study |
| SmHPL1_Seq2R | TAAACGCCAGATCCAGGAGC | [SmHPL (XP_002969700.5)](https://www.ncbi.nlm.nih.gov/protein/XP_002969700.1) | Sequencing | This study |
| SmAOS4_F | CACCATGGCGAGCTTCCTCAGCGATCGATCC | SmAOS4 (XP_002991302.1) | Topo cloning | This study |
| SmAOS4_R | TCACGTCGATGGCGATCGGCGCGAGAGCG | SmAOS4 (XP_002991302.2) | Topo cloning | This study |
| SmAOS4_SeqF | CATCTCCGACCGCTTCAAC | SmAOS4 (XP_002991302.3) | Sequencing | This study |
| SmAOS4_SeqR | CTTGTGGCTCTCGATCACC | SmAOS4 (XP_002991302.4) | Sequencing | This study |
| qSmUbi_F2 | ATCGTCTGGGCAAGAAGGTG | SmUbiquitin (XP_002966475.1) | RT-qPCR | Pratiwi et el. 2017 |
| qSmUbi_F1 | ATACCATCGGCGATTTGAA | SmUbiquitin (XP_002966475.2) | RT-qPCR | Pratiwi et el. 2017 |
| qSmUbi_R2 | GATCCTGATCTTCTCCGGGC | SmUbiquitin (XP_002966475.3) | RT-qPCR | Pratiwi et el. 2017 |
| qSmUbi_R1 | CGCTTACAAGGAAAGCACCT | SmUbiquitin (XP_002966475.4) | RT-qPCR | Pratiwi et el. 2017 |
| SmActin_F | ACTGGGACGACATGGAGAAG | SmActin (XP_002977012.1) | RT-qPCR | Pratiwi et el. 2017 |
| SmActin_R | CGCCTGAATAGCAACGTACA | SmActin (XP_002977012.1) | RT-qPCR | Pratiwi et el. 2017 |
| qSmHPL1_F | CTGGAGGTGATGAGGATCGC | [SmHPL (XP_002969700.4)](https://www.ncbi.nlm.nih.gov/protein/XP_002969700.1) | RT-qPCR | This study |
| qSmHPL1_R | GCGGATGAGAAACTCCCTCC | [SmHPL (XP_002969700.4)](https://www.ncbi.nlm.nih.gov/protein/XP_002969700.1) | RT-qPCR | This study |
| qSmAOS4_F | CGACGAGAGATGGAGTGGTG | SmAOS4 (XP_002991302.2) | RT-qPCR | This study |
| qSmAOS4_R | GGACCACAGCACGTACTTGA | SmAOS4 (XP_002991302.2) | RT-qPCR | This study |
| qSmAOS1_F | GCCCGAGTACCAGAAGATCG | SmAOS1(XP_002978826.1) | RT-qPCR | Pratiwi et el. 2017 |
| qSmAOS1_R | AGAAGACGAGGTTGTGCAGG | SmAOS1(XP_002978826.1) | RT-qPCR | Pratiwi et el. 2017 |
| qSmAOS2_F | CGCAGCTCAAGTCGTATTGC | SmAOS2(EFJ20163.1) | RT-qPCR | Pratiwi et el. 2017 |
| qSmAOS2_R | GACTTCTTCGGCTTGTTGCG | SmAOS2(EFJ20163.1) | RT-qPCR | Pratiwi et el. 2017 |
| qSmAOS3_F | TACCCTTCATCATCGTGGCG | SmAOS3(XP_002978827.1) | RT-qPCR | Pratiwi et el. 2017 |
| qSmAOS3_R | AGAAGACGAGGTTGTGCAGG | SmAOS3(XP_002978827.1) | RT-qPCR | Pratiwi et el. 2017 |
| qSmOPR5-F | AGCTGTGCATGACAAAGGTG | SmOPR5 (XP_002979866.1) | RT-qPCR | Pratiwi et el. 2017 |
| qSmOPR5-R | GGACTGTCCATCTGGGAAGA | SmOPR5 (XP_002979866.1) | RT-qPCR | Pratiwi et el. 2017 |
