# Supplementary material for: Green Leaf Volatile-Burst in Selaginella moellendorffii
Source: Front Plant Sci. 2021 Oct 27;12:731694. doi: 10.3389/fpls.2021.731694 (PMC8578206; doi:10.3389/fpls.2021.731694)
Supplement: Supplementary file 5 [file Table_3.DOCX]

**Supplementary Table 3. Proteins used to construct the phylogenetic tree shown in Figure 7.**

| Plant species | Names in the tree | Accession number |
| --- | --- | --- |
| *Solanum tuberosum* | StDES | CAC28152 |
|  | StAOS3 | CAI30876 |
| *Solanum lycopersicum* | LeDES | AAG42261 |
|  | LeAOS1 | CAB88032 |
|  | LeAOS2 | AAF67141 |
|  | LeAOS3 | AAN76867 |
|  | LeHPL | AAF67142 |
| *Nicotiana tabacum* | NtDES | AAL40900 |
| *Cucumis sativum* | CsHPL1 | AAF64041 |
| *Cucumis melo* | CmHPL | AAK54282 |
|  | CmAOS | AAM66138 |
| *Medicago truncatula* | MtHPL1 | CAC86898 |
|  | MtHPL2 | CAC86899 |
| *Physcomitrella patens* | PpAOS1 | CAC86919 |
|  | PpAOS2 | XP001759629 |
|  | PpHPL | CAC86920 |
| *Psidium guajava* | PgHPL | AAK15070 |
| *Medicago sativum* | MsHPL1 | CAB54847 |
| *Arabidopsis thaliana* | AtHPL | AAC69871 |
|  | AtAOS | CAA63266 |
| *Zea mays* | ZmHPL | AAS47027 |
| *Hordeum vulgare* | HvHPL | CAC82980 |
|  | HvAOS1 | CAB86384 |
|  | HvAOS2 | CAB86383 |
| *Oryza sativum* | OsAOS1 | AY055775 |
| *Musa ascuminata* | MaHPL | CAB39331 |
| *Parthenium argentatum* | PaAOS | CAA55025 |
| *Marchantia polymorpha* | MpAOS1 | BAS32647 |
|  | MpAOS2 | BAS32648 |
| *Klebsordimium nitens* | KnAOS | BAS32649 |
| *Branchiostoma floridae* | BfEAS | ACD88492 |
| *Methylobacterium nodulans* | MnHPL | WP_015932840 |
| *Acropora palmata* | ApAOS | ACD42778 |
| *Adiantum capillus-veneris* | Adiantum_MBC9831437.1 | n.a. |
|  | Adiantum_MBC9844452.1 | n.a. |
|  | Adiantum_MBC9846313.1 | n.a. |
|  | Adiantum_MBC9852615.1 | n.a. |
|  | Adiantum_MBC9855469.1 | n.a. |
|  | Adiantum_MBC9842072.1 | n.a. |
|  |  |  |
| n.a.; not applicable. |  |  |
