# Supplementary material for: Green Leaf Volatile-Burst in Selaginella moellendorffii
Source: Front Plant Sci. 2021 Oct 27;12:731694. doi: 10.3389/fpls.2021.731694 (PMC8578206; doi:10.3389/fpls.2021.731694)
Supplement: Supplementary file 6 [file Table_4.DOCX]

**Supplementary Table 4. The list of CYP74-like genes found in the genome sequences of *S. moellendorffii*.**

| ID | Transcript Name | Accession number | CYP name | Enzyme activity | Reference |
| --- | --- | --- | --- | --- | --- |
| 15402487 | 177201 | EFJ20163.1 | CYP74K3 (SmAOS2) | 13AOS | Pratiwi et al., 2017 |
| 15404149 | 177485 | XP_002979266.1 | CYP74M1 (SmDES1) | 13DES | Gorina et al., 2016 |
| 15407374 | 133317 | XP_002991302.1 | CYP74J1 (SmAOS4) | 13AOS | This study |
| 15408469 | 228572 | XP_002978827.1 | CYP74K2 (SmAOS3) | ? | Pratiwi et al., 2017 |
| 15412037 | 98212 | EFJ26024.1 | CYP74M2 (SmEAS1) | 13EAS | Toporkova et al., 2018 |
| 15412203 | 413157 | [XP_002972649.1](https://www.ncbi.nlm.nih.gov/protein/XP_002972649.1) | CYP74L3 | ? |  |
| 15412249 | 81998 | XP_002964012.2 | CYP74M3 (SmDES2) | 13DES | Gorina et al., 2016 |
| 15415906 | 98717 | [XP_002972651.1](https://www.ncbi.nlm.nih.gov/protein/XP_002972651.1) | CYP74L2 | ? |  |
| 15417742 | 92382 | [XP_002969700.1](https://www.ncbi.nlm.nih.gov/protein/XP_002969700.1) | CYP74L1a (SmHPL1a) | 13HPL | This study |
| n.a. | n.a. |  | CYP74L1b (SmHPL1b) | 13HPL | This study |
| 15422353 | 271334 | XP_002978826.1 | CYP74K1 (SmAOS1) | ? | Pratiwi et al., 2017 |
| 15419374 | 446021 | EFJ14286.1 |  | ? |  |

The genes found with *Selaginella moellendorffii* v1.0 proteome with AtHPL (At4g15440) as the query with E-values of less than 2.4 × 10^-87^are shown.
